# Organophosphate Pesticide Exposure and Work in Pome Fruit: Evidence for the Take-Home Pesticide Pathway

**DOI:** 10.1289/ehp.8620

**Published:** 2006-03-13

**Authors:** Gloria D. Coronado, Eric M. Vigoren, Beti Thompson, William C. Griffith, Elaine M. Faustman

**Affiliations:** 1 Cancer Prevention Research Program, Seattle, Washington, USA; 2 Department of Environmental and Occupational Health Sciences, School of Public Health and Community Medicine, University of Washington, Seattle, Washington, USA

**Keywords:** children of farmworkers, contamination, crops, farmworkers, house dust, occupational exposure, pesticides, take-home pathway, urinary metabolites, vehicle dust, WinBUGS

## Abstract

Organophosphate (OP) pesticides are commonly used in the United States, and farmworkers are at risk for chronic exposure. Using a sample of 218 farmworkers in 24 communities and labor camps in eastern Washington State, we examined the association between agricultural crop and OP pesticide metabolite concentrations in urine samples of adult farmworkers and their children and OP pesticide residues in house and vehicle dust samples. Commonly reported crops were apples (71.6%), cherries (59.6%), pears (37.2%), grapes (27.1%), hops (22.9%), and peaches (12.4%). Crops were grouped into two main categories: pome fruits (apples and pears) and non-pome fruits. Farmworkers who worked in the pome fruits had significantly higher concentrations of dimethyl pesticide metabolites in their urine and elevated azinphos-methyl concentrations in their homes and vehicles than workers who did not work in these crops. Among pome-fruit workers, those who worked in both apples and pears had higher urinary metabolites concentrations and pesticide residue concentrations in dust than did those who worked in a single pome fruit. Children living in households with pome-fruit workers were found to have higher concentrations of urinary dimethyl metabolites than did children of non-pome-fruit workers. Adult urinary concentrations showed significant correlations with both the vehicle and house-dust azinphos-methyl concentrations, and child urinary concentrations were correlated significantly with adult urinary concentrations and with the house-dust azinphos-methyl concentration. The results provide support for the take-home pathway of pesticide exposure and show an association between measures of pesticide exposure and the number of pome-fruit crops worked by farmworkers.

Organophosphate (OP) pesticides are the most widely used pesticides in the United States, and farmworkers are at high risk for exposure. The health effects of acute exposure to pesticides are well characterized. Previous investigations have examined the long-term health effects among workers after an acute or chronic low-level exposure. Studies have reported deficits in verbal and visual attention, motor dexterity, confusion, and lapses in memory, among others (Eskenazi and Maizlish 1988; McConnell et al. 1994; Rosenstock et al. 1991). Others have reported elevated risks for leukemia (Beane Freeman et al. 2005), non-Hodgkin lymphoma (Fritschi et al. 2005), and lung cancer (Beane Freeman et al. 2005), although some studies have not found significant associations (Burns 2005; Reynolds et al. 2005).

The U.S. Environmental Protection Agency (EPA) has published tables of transfer coefficients that estimate the amount of treated foliage that a farmworker contacts while performing occupational tasks on various crops (Science Advisory Council for Exposure 2000). The estimates are based on standard assumptions about protective clothing worn by workers and the absorption rates for pesticides through the skin or inhalation. Higher transfer rates are estimated for workers who thin than for workers who harvest, prune, weed, irrigate, or perform other farm tasks. The extent to which farm task is related to levels of worker pesticide exposure when cross-sectional data are examined remains controversial (Coronado et al. 2004a, 2004b, 2004c; Fenske et al. 2004; Krieger and Zhang 2004).

Items to consider in assessing the relationship of farm tasks and pesticide exposure include transfer coefficients, total amount of pesticides applied, time of pesticide application, and the multiple agricultural crops that farmworkers may work with. Equivalent transfer coefficients are assigned to various orchard crops, such as apples, pears, cherries, and peaches. Data from the U.S. Department of Agriculture (USDA) (2000) show that differing crops have varying amounts of pesticides applied. Farmworkers generally work in a variety of crops during a given growing season, and it remains unclear how work in multiple agricultural crops influences overall worker exposure.

Growing research interest is seen in the levels and patterns of pesticide exposure among children of farmworkers. This emphasis was driven by a report of the National Research Council (1993) expressing concern about pesticide residues on food. Some studies have identified possible risks for the development of cancers, birth defects, and abnormal reflexes among children and neurologic impairments among adults and children (Blain 2001; Guillette et al. 1998; Kirkhorn and Schenker 2002; Mills and Yang 2003; Rohlman et al. 2005; U.S. General Accounting Offices 2000; Young et al. 2005).

Pesticide exposure in children is of concern because of the way in which exposure occurs. Exposure is not always direct; it is generally believed to occur from pesticides brought to the home through the take-home pathway of farmworkers (Thompson et al. 2003). Such paraoccupational exposure is important because of children’s unique behaviors, such as greater amounts of time spent on floors where pesticides accumulate, increased likelihood of dermal exposure from wearing minimal clothing during the summer spray season, and increased likelihood of pesticide ingestion from hand-to-mouth behavior (Mills and Zahm 2001).

The importance of agricultural task was shown by Fenske et al. (2000), who reported that children of pesticide applicators had higher urinary concentrations of dialkylphosphates than do children of nonagricultural workers living in the same community (based on creatinine-adjusted spray-season estimates). Lambert et al. (2005) have reported crop-specific information showing that children whose parents worked in pear orchards had higher concentrations of pesticide metabolites in their urine than did children whose parents worked in berries or cherries. Other studies have shown that children of agricultural workers have higher exposures than children of nonagricultural workers (Loewenherz et al. 1997; Lu et al. 2000) and that pesticide metabolite levels in children’s urine correlate with pesticide metabolite levels in adults’ urine within the same household (Curl et al. 2002). A limited number of previous investigations have examined households for the presence of pesticide residues in dust samples where children are thought to be at risk of exposure (Coronado et al. 2004c; Curwin et al. 2005; Fenske et al. 2002; Lu et al. 2000; McCauley et al. 2003; Shalat et al. 2003).

Using a large sample of farmworkers from several agricultural communities in eastern Washington State, we examined the association between work in specific agricultural crops and levels of OP pesticide exposure among adult workers and children living in the same household. We aimed to test the hypothesis that the take-home pathway results in children’s exposure to pesticides.

## Materials and Methods

### Setting

A study that tested a culturally appropriate intervention to interrupt the take-home pathway of pesticide exposure provides the data for this report. The setting, study design, study participants, and survey procedures have been described previously (Thompson et al. 2003). Briefly, the study took place in the Yakima Valley of Washington State. An estimated 50,000 people in the region work in agriculture; the primary crops are apples, grapes, pears, cherries, hops, and peaches (USDA 2000). Approximately 50% of the area population is Hispanic, and most work in agriculture. For most crops cultivated in the Yakima Valley, fieldwork is done by hand.

In Washington State during 1999 (the year in which these data were collected), 172,000 acres of farmland were dedicated to apple production; substantial acreage was dedicated to the production of pears (24,400 acres), cherries (18,000 acres), peaches (2,500 acres), hops (25,076 acres), and grapes (41,000 acres) (USDA 2000). Yakima County ranked first among Washington State counties in number of acres dedicated to the production of apples (75,264 acres), pears (10,190 acres), cherries (6,129 acres), peaches (1,438 acres), and hops (20,061 acres) and second in the acres dedicated to grape production (15,529 acres) (USDA 2004).

### OP pesticide use in Washington State

One of the most commonly used pesticides in the Yakima Valley is azinphos-methyl. This is a broad-spectrum insecticide registered for use in the control of many insect pests on a wide variety of fruit, vegetable, nut, and field crops as well as on ornamental plants, tobacco, and forest and shade trees. Azinphos-methyl is classified by the U.S. EPA as having level I toxicity. It is reported to be highly toxic through inhalation, dermal absorption, ingestion, and eye contact (Extension Toxicology Network 1996). The current reentry interval for azinphos-methyl applied to apples, pears, and peaches is 14 days and for cherries, 15 days (USDA 2003), during which time, only workers wearing protective equipment are allowed in the fields. In 1999, the reentry interval for azinphos-methyl was extended from 48 hr to 14 days. It is unclear to what extent the shorter reentry interval was in practice during the 1999 season. For each annual crop season, applications are limited to 8 pounds per acre for apples, 6 pounds per acre for pears, and 3 and 4.5 pounds per acre for cherries and peaches, respectively (Bayer CropScience LP 2003). Data from the voluntary Washington Agricultural Statistics survey show that in 1999 an estimated 309,300 pounds of azinphos-methyl were applied to Washington State apple orchards and 33,000 pounds, 17,500 pounds, and 2,000 pounds were applied to pear, cherry, and peach orchards, respectively (USDA 2000). Azinphos-methyl is generally not used in hop or grape production (USDA 2001; Washington Association of Wine Growers 2004).

Other OP pesticides were also used on Yakima Valley crops in 1999 (USDA 2000): Phosmet was applied to Washington State apples and pears in estimated quantities of 46,000 pounds and 20,600 pounds, respectively; malathion was applied to apples and peaches in quantities of 22,300 pounds and 1,500 pounds, respectively; methyl-parathion was applied to apples and pears in quantities of 17,100 pounds and 1,400 pounds, respectively; and chlorpyrifos was applied to apples, pears, peaches, cherries, and grapes in quantities of 250,900 pounds, 28,300 pounds, 1,300 pounds, 20,600 pounds, and 8,000 pounds, respectively.

### Questionnaire

An in-person interview was conducted with the farmworkers. The main questionnaire was a 73-item instrument that included nine sections. Workers were asked whether or not they had worked with apples, pears, peaches, cherries, hops, or grapes in the previous 3 months, and to name any additional crops. Workers were also asked whether in the previous 3 months they had performed the following agricultural job tasks: harvesting or picking; pruning; loading; packing; sorting or grading plants, fruits, or vegetables; planting or transplanting; weeding; thinning; irrigating; mixing or loading farm chemicals; spraying or applying pesticides; or other tasks. Before implementation, the questionnaire was translated into Spanish, piloted among farmworkers, and reviewed and edited by members of a community advisory board. The questionnaire and all study procedures were reviewed and approved by the Fred Hutchinson Cancer Research Center. All adult participants and parents of child participants gave informed consent to participate in the study.

### Survey procedures

Recruitment procedures have been described previously (Thompson et al. 2003). Briefly, individuals who worked in agriculture were recruited using an in-person survey of randomly selected households conducted for another study. Additional workers were recruited from labor camps and from areas in the community known to have a large concentration of agricultural workers. A total of 571 households were surveyed; 218 of these households had age-eligible children (2–6 years of age) and agreed to enroll in the specimen collection aspect of the study. This subgroup forms the sample basis for this report.

Among eligible households (those with a farmworker and age-eligible child), an adult respondent and study child were identified (Thompson et al. 2003). We collected urine samples from the adult farmworker and study child as well as dust samples from selected areas of the home and the vehicle used to commute to and from work. Samples were collected between June and October 1999.

### Specimen collection and laboratory analysis

Procedures for the urine and dust collection and laboratory analysis have been described in detail elsewhere (Curl et al. 2002). Two or three spot urine samples were collected. Each collection was separated by a minimum of 3 days and collected within a 2-week period, with the first collection occurring at the interview. For each individual, equal volumes of each urine sample were combined before specimen analysis. This provided an estimate of pesticide exposure within the 2-week period of assessment.

Urine samples were analyzed using gas chromatographic procedures for the presence of five dialkylphosphate compounds produced by the metabolism of most OP pesticides: dimethylphosphate (DMP), dimethylthio-phosphate (DMTP), dimethyldithiophosphate (DMDTP), diethylphosphate, and diethyl-thiophosphate. The limits of detection for these compounds were 7.2 μg/L, 1.1 μg/L, 0.65 μg/L, 2.9 μg/L, and 1.2 μg/L, respectively.

Dust samples were collected from homes and commuter vehicles using a Nilfisk vacuum cleaner unit (model GS-80; Nilfisk of America, Malvern, PA), and sampling took place within 4 weeks of the interview. Selection of the area to be vacuumed was determined by asking the parent or adult participant where the child played most frequently. The size of the area vacuumed depended on the floor type and ranged from a 1 m × 1 m area for plush carpets to a 2 m × 2 m area for hard or smooth floors. Foot wells from both the front and back of cars (and only the front of trucks) were vacuumed. Mats were not removed before vacuuming. Dust samples were analyzed using gas chromatographic procedures for the presence of four dimethyl OP pesticides (azinphos-methyl, malathion, methyl-parathion, and phosmet) and two diethyl OP pesticides (chlorpyrifos and diazinon). The limits of detection for these pesticides residues and the percentage of analyzed samples that contained detectable levels are shown in Table 1. For this report, we limit our analyses of the dust samples to the azinphos-methyl residues because in a large number of samples other pesticides were below detection levels. Because azinphos-methyl is a dimethyl OP pesticide, we limited our analyses of the urine samples to the dimethyl metabolites.

### Statistical and classification methods

Apples and pears are classified as pome fruit: a fleshy fruit having several seed chambers. Peaches and cherries are stone fruits, containing a single seed or pit. Grapes and hops are vine/trellis and bunch/bundle crops, respectively. We grouped workers into two categories based on crop type. Pome-fruit workers were those who worked in apples and/or pears and possibly peaches, cherries, grapes, hops, and other crops. Non-pome-fruit workers worked only in peaches, cherries, grapes, hops, or other crops. We grouped pears with apples because they have similar harvest seasons, and both are chemically and hand-thinned during the pesticide spray season (June through August). The rates and types of pesticide application are similar for both crops.

We present the frequencies of detection and estimated geometric mean (GM) concentrations and geometric standard deviations of adult and child urinary dimethyl metabolites and azinphos-methyl residues in vehicle and house-dust samples. Concentrations for the urinary samples are presented in units of micrograms per liter and are not creatinine adjusted. Concentrations for the dust samples are presented in units of micrograms per gram.

Quantile–quantile plots (not shown) demonstrate that the concentrations of the urinary metabolites and of pesticide residues in dust were approximately normally distributed after a log-transformation. Because some samples had values below the limits of detection, we modeled the missing data in a multivariate normal hierarchical Bayesian simulation model with conjugate noninformative priors using WinBUGS (Windows-based Bayesian inference Using Gibbs Sampling) (Spiegelhalter et al. 2003 ). The data below the limits of detection were treated as left censored data with an upper cutoff at the limit of detection (Griffith et al. 2002). The statistical model assumed a common variance/covariance structure while allowing for a shift in the means for different farmworker classifications. After a burn-in simulation run, we performed 250,000 simulations. Confidence limits for the log-normal parameters (the 95% posterior predictive probability intervals) were estimated. To examine differences in the means of farmworker groups, at each simulation we compared the means to see which were larger. These comparisons were recorded and summed, and we report the probability that one GM was greater than another.

## Results

In this study, 571 (89.6%) of the 627 farm-workers identified for the study were interviewed. A small percentage of households (3.8%) could not be contacted after at least five visits, and 6.6% of eligible farmworkers refused to participate, giving an overall response rate of 89.6%, or 93.1% of the known eligibles. Of the 571 respondents, 231 households (40.5%) included children 2–6 years of age. Of these, 218 households were available for sample collection. The total number agreeing to provide urine samples included 213 adult farmworkers (92.2% of those eligible) and 211 children (91.3% of those eligible). House-dust samples were collected from 210 homes, and of the households with vehicles (*n* = 207), 205 (99.0%) allowed us to collect vehicle dust. For 54 homes and 15 vehicles, insufficient masses of dust were collected for analysis; thus, pesticide residue analysis was conducted on 156 house-dust samples and 190 vehicle-dust samples.

Three-quarters of the farmworkers in our study sample reported working in apples within the 3 months before being interviewed (Table 2). More than one-third worked in pears, and nearly two-thirds worked in cherries. Approximately one-tenth of workers worked in peaches and nearly one-quarter worked in grapes or hops. Fewer than one-third of the respondents worked in other crops, which included asparagus, apricots, plums, peppermint, corn, and onions.

When farmworkers reported working in multiple crops, apples were the crop with the greatest overlap with other crops. All of the peach workers and all but two pear workers had also worked in apples. The percentages of cherry, grape, hop, and other crop workers who had also worked in apples were 80.8, 69.5, 62.0, and 61.9%, respectively.

Those who worked in pome fruits were slightly older than those who worked in non-pome fruits (Table 3). Pome-fruit workers, on average, had fewer years of education and had lower household incomes than did non-pome-fruit workers; those working in both apples and pears on average had the lowest education and income levels. The groups were similar in marital status. Birthplace differed slightly by crop category. Approximately one-fifth of pome-fruit workers reported having worked in agriculture for 20 or more years; the proportion was about one-fourth for non-pome-fruit workers. Most adult participants were male and completed the survey in Spanish.

Eight of the farmworkers had DMP concentrations that were orders of magnitude higher than other study participants and ranged from 3,780 to 12,000 μg/mL (the remaining study participants’ concentrations ranged from less than the limit of detection to 100 μg/mL). All eight farmworkers reported working in apples, and half had also worked in pears; seven had worked as thinners. We performed our analysis with and without the data from these farmworker households. The two analyses showed similar relationships between urinary metabolite and dust concentrations and crop category; however, those households were influential in estimating the GMs and the geometric standard deviations. Including them in the analysis made it unclear whether the crop effect was primarily a result from these eight households, or whether there was a crop effect among all who worked in pome fruit. By excluding them, we could show that the association between crop category and metabolite and dust concentrations was present among the rest of the population. Therefore, measurements taken from these eight households are not represented in Tables 4–8; the results reported in these tables are based on 210 farmworker households.

Examining the dimethyl urinary metabolites among workers who did or did not work in pome fruits, we observed differences in the frequency of detection and in the metabolite concentrations (Table 4). Workers who reported working in pome fruit had higher concentrations of dimethyl metabolites than did non-pome-fruit workers; GM concentrations were 2.4-fold, 3.5-fold, and 2.9-fold higher for DMP, DMTP, and DMDTP, respectively. Among pome-fruit workers, those who worked in both apples and pears had the highest dimethyl metabolite concentrations.

Children in our study had patterns of exposure that were similar to those in adults (Table 4). Children who lived in households with a farmworker who worked in pome fruit had greater frequency of detection of dimethyl metabolites than did children living in households with a non-pome-fruit worker. The frequency of detection for DMP, for example, was 7.1% for children living in households with non-pome-fruit workers and 22.5% for children living with pome-fruit workers. GM concentrations of DMP, DMTP, and DMDTP were 2.6-fold, 1.7-fold, and 1.5-fold higher, respectively, for children who lived in households with a pome-fruit worker. Among children living with a pome-fruit worker, those who lived in households with an apple and pear worker had higher dimethyl metabolite concentrations than those who lived in households with an apple or pear worker only.

Differences between the crop worker groups are seen in the concentrations of azinphos-methyl residue in the dust samples (Table 5). More than 90% of those who worked in pome fruit had detectable azinphos-methyl in their vehicles and homes, compared with only slightly more than 60% for the non-pome-fruit workers. Among pome-fruit workers, those who worked in both apples and pears had the greatest percentage detection and higher concentrations of azinphos-methyl in their house and vehicle dust. Those who worked in pome fruit had GM concentrations of azinphos-methyl in their vehicle and house dust that were 6.8-fold and 4.6-fold greater, respectively, than those for farmworkers who did not work in pome fruit.

The estimated correlations for the dimethyl urinary metabolite concentrations and the azinphos-methyl concentrations in house and vehicle dust that we observed are given in Table 6. Within both the adult and the child urine samples, there were statistically significant high positive correlations between the dimethyl metabolite concentrations, particularly for DMTP and DMDTP concentrations. The vehicle- and house-dust concentrations of azinphos-methyl were also highly correlated. Urine samples of children and adults living in homes with elevated levels of dust indicate exposure to higher levels of pesticides; adult urine DMP and DMTP concentrations showed significant correlations with both the vehicle- and house-dust azinphos-methyl concentrations, and child urine DMP and DMTP concentrations were correlated significantly with the house-dust azinphos-methyl concentration.

When we examined differences in urinary metabolite concentrations among those who did and did not perform thinning within pome-fruit and non-pome-fruit worker categories, we found no remarkable differences between the groups (Table 7). Similarly, there were no notable differences in concentrations of azinphos-methyl and vehicle and dust samples between thinners and nonthinners when categorized by work in pome fruit (Table 8).

## Discussion

We assessed OP pesticide exposure among farmworkers based on whether on not they worked in pome-fruit crops. Those who worked in pome fruit had higher concentrations of OP pesticide metabolites in their urine and higher concentrations of azinphos-methyl residues in dust collected from their homes and vehicles. The increased presence of pesticide-laden dust probably contributed to the pesticide exposure of children in the household.

Previously, we reported higher proportions of urine samples with detectable levels of the OP pesticide urinary metabolite DMTP from children of farmworkers who reported thinning, compared with urine samples from children of nonthinners. We also reported higher proportions of detectable azinphos-methyl in vehicle and house dust among workers who reported thinning (Coronado et al. 2004c). The present findings suggest that agricultural crop was an important factor that was not considered in our previous study; 91.4% of thinners reported having worked in pome fruits in the 3 months before the survey. After controlling for work in pome-fruit crops, our data revealed no significant differences between workers who did or did not report thinning, for both percent detection and concentration measurements.

The higher urinary metabolite levels found in pome-fruit workers matched pesticide use patterns in Washington State. Data from the Washington Agricultural Statistics survey show that in 1999 the rate of application of azinphos-methyl was higher in apples (1.8 lb/acre) than in pears (1.4 lb/acre), cherries (1.0 lb/acre), or peaches (0.8 lb/acre) (USDA 2000); thus, our data appear to show higher exposure levels among those who worked in crops with the highest pesticide applications.

Particularly noteworthy is the distinct gradient we see in the azinphos-methyl concentrations in vehicle and house dust. Samples collected from households of farm-workers who worked in both apples and pears had the higher pesticide concentrations than did samples collected from households of farmworkers who worked in only one pome fruit (Table 5). Samples collected from households of farmworkers who did not work with pome fruit had the lowest concentrations. The association between higher adult and child urinary OP pesticide metabolite concentrations and work with increasing numbers of pome fruit was also observed (Table 4). These findings suggest the potential for cumulative pesticide exposure among workers performing tasks on multiple crops.

We found no association within worker groups between pesticide and metabolite concentrations and self-reported information about the recent application of pesticides in the workplace. This suggests that pesticides that accumulate in the home environment may account in part for the urinary metabolite levels of adults and children, and that pome-fruit workers are more likely to track home pesticides. The high positive correlation between azinphos-methyl levels in house and vehicle dust that we observed along with the statistically significant positive correlations between the dust concentrations and urinary metabolite concentrations provides perhaps the strongest evidence for the take-home pathway (Table 6).

The Centers for Disease Control and Prevention (CDC 2005) reported that the GM concentration of the OP pesticide urinary metabolite DMTP for adults 20–59 years of age in the general population is 1.47 μg/L averaged across a year. We observed concentrations three times higher in the spray season for farmworker adults of similar ages who did not work in pome-fruit crops (GM = 4.4 μg/L) and concentrations > 10 times higher for those who did (GM = 15.3 μg/L). Although the CDC report provides no data for children in the same age group as our study, it does give a GM urinary metabolite concentration of 2.95 μg/L for DMTP in children 6–11 years of age. Our findings for children 2–6 years of age are 1.2 times higher than this for those in households of farmworkers who did not work in pome fruits (GM = 3.5 μg/L) and > 2.1 times higher for those in households of farmworkers who did work in pome fruits (GM = 6.2 μg/L). Because our children were younger, they may have higher concentrations of metabolites than do older children, as has been reported in a limited number of previous investigations on the topic (Loewenherz et al. 1997; Shalat et al. 2003).

Few previous pesticide exposure investigations have examined household dust. We found higher concentrations of azinphos-methyl in house and vehicle dust among workers who reported having worked in the pome fruits versus farmworkers who did not report working in these fruits. Our GM concentration of azinphos-methyl in house dust (0.79 μg/g) among pome-fruit workers is comparable with that reported for agricultural workers by Lu et al. (2000) (median concentration = 1.0 μg/g combined value for pesticide applicators and farmworkers in pome-fruit–growing region in Washington State) and Shalat et al. (2003) (median concentration = 0.51 μg/g for homes in an agricultural community near the U.S.–Mexico border).

Our analyses support the notion that children of farmworkers are exposed through pesticides that are tracked into homes. Exposures among adult workers and children living in the same household varied with the agricultural crop in which the adult worked. This finding would not be expected if dietary ingestion of pesticides or home use of pesticides were primary sources of exposure because these factors are unlikely to be related to work in given agricultural crops. Our analyses, however, indicate that pesticide residues found in house and vehicle dust were significantly greater for workers who worked in pome fruit than for those who did not, providing evidence for increased exposure to pesticides that are subsequently tracked from the fields to worker vehicles and homes. Moreover, among the pome-fruit workers, we observed higher concentrations of azinphos-methyl in vehicle dust than in house dust, consistent with expectations that pesticides are carried from work-places to vehicles and eventually homes on workers’ clothing, hats, and boots.

Analyses of urine from children of farm-workers reported by other studies also provide support for a take-home pathway. Lu et al. (2000) showed that median DMTP concentrations in agricultural children were four times higher than those of nonagricultural children. A study by Koch et al. (2002) collected urine samples from 44 children on a biweekly basis over a period of 21 months. Data from the study show that children had higher GM concentrations of combined dialkylphosphates during the spray months compared with the nonspray months. These findings support the take-home pathway but do not discount the contributions of other pathways. Our data set was collected shortly after the spray season. In a previous analysis of this data, Curl et al. (2002) demonstrated a strong correlation between urinary dimethyl metabolite concentrations of adult farmworkers and children living in the same household, a finding that would be expected with the take-home pathway. Studies that examined pesticide residues in dust samples also provide support for the take-home pathway. Separate studies conducted by Lu et al. (2000) and Fenske et al. (2000) report higher median house-dust concentrations of azinphos-methyl in homes of agricultural families compared with nonagricultural families, and McCauley et al. (2003) showed that house-dust concentrations rose with increasing numbers of agricultural workers in a household.

### Limitations

This study has some limitations. It is possible that some urine and dust samples were collected relatively late in the spray season. Although we report higher urinary metabolite concentrations for those who worked in crops where higher amounts of pesticides were used, the late-season collection of urine samples may have increased the likelihood of detecting metabolites from pesticides used on crops that are harvested late in the season (e.g., apples and pears). Nevertheless, we relied on local knowledge and previous research conducted in the area to determine when to begin collection (Simcox et al. 1999).

Apart from the possibility that the late-season collection may have attenuated our reported exposure estimates, it is likely that our urinary metabolite concentrations underestimate levels during peak exposures. Information from the Washington State Tree Fruit Research Commission shows that temperature readings for the spring of 1999 were much lower than normal and resulted in the recommendation to growers to cancel or delay the first several sprayings of the season (Koch et al. 2002). The first application of OP pesticide for the 1999 season is reported to have been in April, and the number of applications in that season was reduced; thus, the levels of OP pesticide urinary metabolites in our samples may have been lower than for other years. Our urinary metabolite estimates may have been further attenuated because peak excretion of metabolites occurs relatively quickly (24–48 hr after exposure) (Feldmann and Maibach 1974) and we did not time our collections according to spray events. Despite these potential limitations, we were able to find significant differences (based on crop category) both in urinary OP metabolite concentrations in adults and children and in pesticide concentrations in home and vehicle dust.

Questions asked about work in the past 3 months resulted in a high percentage of workers reporting work in multiple crops; thus, our ability to discern differences in exposure levels associated with given crops was limited. An additional limitation of our assessment of occupational pesticide exposure is that we did not include number of hours worked per week. This factor could account for a substantial fraction of pesticide exposures and could explain differences in urinary pesticide metabolite concentrations. Although we do not believe this limits the conclusions we can draw from our results, further investigations on this topic would benefit by collection of this variable and its inclusion in analysis.

Farmworkers as a group are difficult populations to assess (Kamel et al. 2001; Zahm and Blair 2001). The strength of this study is the large sample size and the substantial variation in types of agricultural crops reported in our sample. Moreover, our data reflect the real-life experience of farmworkers in that most work in multiple crops. Two unique features of our statistical analyses techniques were strengths. First, we were able to concurrently examine the relationships between adult and child urinary OP pesticide concentrations and pesticide residues in house and vehicle dust based on crop and job task categories, whereas previous investigations have generally considered only two-way comparisons. Second, we used a modeling technique to estimate values that were below the limit of detection (Griffith et al. 2002); this offered greater predictive power compared with some previous investigations that have assigned a single value to data points that are below the limit of detection or have considered only data points above the limit of detection. Previous investigations have recruited farmworkers from a select number of farms or community organizations (Fenske et al. 2000; Koch et al. 2002; Loewenherz et al. 1997; Lu et al. 2000; Mills and Zahm 2001; Simcox et al. 1999). Growers who volunteer their farms for participation in studies may promote more protective work practices or apply fewer applications of pesticides, thus potentially biasing collected data. Our household recruitment process attempted to minimize this bias.

## Conclusions

Two general conclusions can be drawn from our findings. First, our findings demonstrate the potential for increased pesticide exposure among workers performing tasks on multiple crops. This is shown by the elevated urinary OP metabolite concentrations (in adults and children) and pesticide concentrations in dust samples in those who worked in both applies and pears, compared with those who worked in a single pome fruit. Second, our findings support the notion that pesticides are tracked into homes of workers, where children are exposed. This is demonstrated by the correlation in the quantity and type of pesticides found in the home and accompanying vehicle, the finding that adults had higher urinary OP pesticide metabolites than did children, and the direct correlation between the concentration of pesticides in house dust and the concentrations of urinary OP metabolites in both adults and children.

## Figures and Tables

**Table 1:** Limits of detection of pesticide residues in dust (μg/g) and percentages of analyzed vehicle (*n* = 190) and house-dust samples (*n* = 156) containing detectable levels of pesticide residue.

	Azinphos-methyl	Phosmet	Malathion	Methyl-parathion	Chlorpyrifos	Diazinon
Limit of detection (μg/g)	0.08	0.08	0.13	0.11	0.11	0.11
Vehicle dust (%)	87	16	12	22	18	2
House dust (%)	85	15	13	14	26	4

**Table 2:** Crops in which study participants (*n* = 218) reported performing agricultural job tasks within the previous 3 months.

	Apples	Pears	Peaches	Cherries	Grapes	Hops	Other
Apples	156^a^						
Pears	79	81					
Peaches	27	24	27				
Cherries	105	62	17	130^a^			
Grapes	41	18	6	32^a^	59		
Hops	31^a^	16	7	24	20	50	
Other	39	21	11	36	12	13	63^a^

a No answer was recorded for three different farmworkers as to whether or not they worked in apples, cherries, or other crops, respectively. For these cross-tabulations, *n* = 217.

**Table 3:** Demographic characteristics of study participants (%): selected adult farmworkers with a child 2–6 years of age in the household by pome/non-pome crop classification (*n* = 217^a^).

Characteristic	Non-pome (*n* = 59)	Pome (*n* = 158)	One pome (*n* = 79)	Two pome (*n* = 79)
Age (years)				
18–24	15.3	8.9	7.6	10.1
25–34	47.5	41.1	46.8	35.4
35–49	23.7	28.5	24.1	32.9
≥50	5.1	5.1	6.3	3.8
Not reported	8.5	16.5	15.2	17.7
Education				
< 4th grade	25.4	32.3	27.8	36.7
5th through 8th	35.6	41.1	41.8	40.5
9th through 12th	32.2	21.5	22.8	20.3
≥High school graduate	6.8	5.1	7.6	2.5
Annual household income (US$)				
< 10,000	18.6	21.5	19.0	24.1
10,000 < 15,000	22.0	29.1	29.1	29.1
15,000 < 25,000	49.2	37.3	38.0	36.7
≥25,000	10.2	10.1	11.4	8.9
Not reported	0.0	1.9	2.5	1.3
Marital status				
Married or living as married	86.4	88.6	91.1	86.1
Separated or divorced	3.4	2.6	2.5	2.5
Never married	10.2	8.2	6.3	10.1
Other	0.0	0.6	0.0	1.3
Birthplace				
Mexico	83.1	94.9	93.7	96.2
United States	15.3	3.8	5.1	2.5
No. of years working in agriculture				
< 10	45.8	48.1	53.2	43.0
10– < 20	28.8	31.0	22.8	39.2
≥20	25.4	20.9	24.1	17.7
Male sex	57.6	67.7	64.6	70.9
Interview in Spanish	86.4	94.3	92.4	96.2

a Total *n* = 217 because of one pome classification missing value.

**Table 4:** Frequency of detection and estimated GM concentrations of dimethyl urinary metabolites among adult farmworkers and their children, by agricultural crop (*n* = 210).

Metabolite and crop	Detection^a^ (%)	Estimated^b^ GM (μg/L)	Estimated^b^ GSD	*p*(pome_GM_ ≤ non-pome_GM_)
Adult DMP			5.96 (4.02–10.74)	
Non-pome fruit	8.8	0.71 (0.20–1.68)		0.017
Pome fruit	20.4	1.72 (0.80–2.89)		
Apples or pears	14.7	1.19 (0.42–2.45)		
Apples and pears	26.4	2.22 (0.97–4.00)		
Adult DMTP			4.48 (3.90–5.29)	
Non-pome fruit	86.0	4.35 (2.92–6.47)		0.000
Pome fruit	96.6	15.34 (12.02–19.54)		
Apples or pears	94.7	13.42 (9.55–18.93)		
Apples and pears	98.6	17.52 (12.41–24.83)		
Adult DMDTP			6.72 (5.34–8.91)	
Non-pome fruit	36.8	0.47 (0.26–0.81)		0.001
Pome fruit	61.2	1.37 (0.97–1.90)		
Apples or pears	60.0	1.19 (0.74–1.88)		
Apples and pears	62.5	1.58 (0.98–2.49)		
Child DMP			2.84 (2.28–3.91)	
Non-pome fruit	7.1	1.34 (0.59–2.39)		< 0.001
Pome fruit	22.5	3.53 (2.40–4.65)		
Apples or pears	18.9	3.06 (1.87–4.38)		
Apples and pears	26.0	3.96 (2.54–5.49)		
Child DMTP			3.61 (3.19–4.20)	
Non-pome fruit	78.6	3.54 (2.50–4.98)		0.003
Pome fruit	91.2	6.18 (5.00–7.61)		
Apples or pears	93.2	5.76 (4.29–7.76)		
Apples and pears	89.0	6.61 (4.89–8.90)		
Child DMDTP			4.83 (3.90–6.30)	
Non-pome fruit	41.1	0.65 (0.39–1.03)		0.061
Pome fruit	46.3	0.98 (0.71–1.30)		
Apples or pears	40.5	0.88 (0.58–1.32)		
Apples and pears	52.1	1.08 (0.71–1.59)		

GSD, geometric SD. Ranges are posterior predictive probability intervals.

a Based on the number of samples analyzed: non-pome, adult *n* = 57, child *n* = 56; apples or pears, adult *n* = 75, child *n* = 74; apples and pears, adult *n* = 72, child *n* = 73.

b Based on the total number of samples: non-pome, *n* = 59; apples or pears, *n* = 75; apples and pears, *n* = 75; missing fruit classification, *n* = 1.

**Table 5:** Frequency of detection and estimated GM concentrations and geometric standard deviations (GSDs) of azinphos-methyl residues in vehicle and house dust (*n* = 210).

Pesticide and crop	Detection^a^ (%)	Estimated^b^ GM (μg/g)	Estimated^b^ GSD	*p* (pome_GM_ ≤ non-pome_GM_)
Vehicle azinphos-methyl			4.65 (3.97–5.61)	
Non-pome fruit	63.5	0.17 (0.11–0.26)		< 0.001
Pome fruit	95.4	1.16 (0.89–1.51)		
Apples or pears	94.1	0.78 (0.54–1.11)		
Apples and pears	96.8	1.79 (1.24–2.58)		
House azinphos-methyl			3.55 (3.07–4.25)	
Non-pome fruit	62.5	0.17 (0.11–0.25)		< 0.001
Pome fruit	92.7	0.79 (0.63–1.00)		
Apples or pears	90.7	0.59 (0.43–0.82)		
Apples and pears	94.6	1.05 (0.76–1.45)		

Ranges are posterior predictive probability intervals.

a Based on the number of samples analyzed: non-pome, vehicle *n* = 52, house *n* = 40; apples or pears, vehicle *n* = 68, house *n* = 54; apples and pears, vehicle *n* = 62, house *n* = 55.

b Based on the total number of samples: non-pome, *n* = 59; apples or pears, *n* = 75; apples and pears, *n* = 75; plus one sample with missing fruit classification.

**Table 6:** Correlation matrix of dimethyl phosphate urinary metabolite concentrations and azinphos-methyl residue concentrations in vehicle and house dust (*n* = 210).

	Adult			Child			Azinphos-methyl	
Metabolite or pesticide	DMP	DMTP	DMDTP	DMP	DMTP	DMDTP	Vehicle	House
Adult DMP	1.00							
Adult DMTP	0.51*	1.00						
Adult DMDTP	0.35*	0.73*	1.00					
Child DMP	0.20	0.12	0.12	1.00				
Child DMTP	0.21*	0.34*	0.22*	0.53*	1.00			
Child DMDTP	0.13	0.34*	0.37*	0.39*	0.81*	1.00		
Vehicle azinphos-methyl	0.28*	0.22*	0.13	0.10	0.15	0.09	1.00	
House azinphos-methyl	0.32*	0.25*	0.09	0.25*	0.24*	0.16	0.52*	1.00

* Statistically significant: 95% posterior predictive probability interval does not include 0.0.

**Table 7:** Frequency of detection and estimated GM concentrations of dimethyl urinary metabolites among adult farmworkers and their children, by agricultural crop (*n* = 210).

Metabolite and pome versus thin	Detection^a^ (%)	Estimated^b^ GM (μg/L)	*p*(thin_GM_ ≤ non-thin_GM_)
Adult DMP			
Non-pome/non-thin	8.9	0.70 (0.17–1.82)	0.641
Non-pome/thin	8.3	0.49 (0.05–2.56)	
Pome/non-thin	20.7	1.93 (0.59–4.63)	0.669
Pome/thin	20.3	1.55 (0.67–2.76)	
Adult DMTP			
Non-pome/non-thin	84.4	3.84 (2.45–5.99)	0.111
Non-pome/thin	91.7	7.01 (2.96–16.50)	
Pome/non-thin	96.6	15.07 (8.74–26.03)	0.470
Pome/thin	96.6	15.43 (11.76–20.22)	
Adult DMDTP			
Non-pome/non-thin	37.8	0.46 (0.23–0.85)	0.443
Non-pome/thin	33.3	0.50 (0.14–1.68)	
Pome/non-thin	65.5	1.71 (0.81–3.53)	0.752
Pome/thin	60.2	1.30 (0.88–1.87)	
Child DMP			
Non-pome/non-thin	6.7	1.15 (0.44–2.26)	0.335
Non-pome/thin	9.1	1.53 (0.37–4.13)	
Pome/non-thin	21.4	3.37 (1.73–5.65)	0.477
Pome/thin	22.7	3.42 (2.25–4.62)	
Child DMTP			
Non-pome/non-thin	77.8	3.62 (2.45–5.30)	0.602
Non-pome/thin	81.8	3.24 (1.49–6.95)	
Pome/non-thin	85.7	7.52 (4.63–12.18)	0.813
Pome/thin	92.4	5.90 (4.67–7.45)	
Child DMDTP			
Non-pome/non-thin	40.0	0.60 (0.34–1.02)	0.336
Non-pome/thin	45.5	0.77 (0.26–2.11)	
Pome/non-thin	53.6	1.18 (0.62–2.20)	0.751
Pome/thin	44.5	0.93 (0.66–1.28)	

Ranges are posterior predictive probability intervals.

a Based on the number of samples analyzed: non-pome/non-thin, adult *n* = 45, child *n* = 45; non-pome/thin, adult *n* = 12, child *n* = 11; pome/non-thin, adult *n* = 29, child *n* = 28; pome/thin, adult *n* = 118, child *n* = 119.

b Based on the total number of samples: non-pome/non-thin, *n* = 47; non-pome/thin, *n* = 12; pome/non-thin, *n* = 29; pome/thin, *n* = 121; plus one sample with missing fruit classification.

**Table 8:** Frequency of detection and estimated GM concentrations of azinphos-methyl residues in vehicle and house dust (*n* = 210).

Pesticide and crop	Detection^a^ (%)	Estimated^b^ GM (μg/g)	*p*(thin_GM_ ≤ non-thin_GM_)
Vehicle azinphos-methyl			
Non-pome/non-thin	63.4	0.18 (0.11–0.29)	0.677
Non-pome/thin	63.6	0.14 (0.05–0.36)	
Pome/non-thin	96.0	0.96 (0.53–1.75)	0.242
Pome/thin	95.2	1.22 (0.91–1.63)	
House azinphos-methyl			
Non-pome/non-thin	58.1	0.14 (0.09–0.22)	0.087
Non-pome/thin	77.8	0.27 (0.12–0.61)	
Pome/non-thin	91.3	0.65 (0.39–1.09)	0.201
Pome/thin	93.0	0.83 (0.64–1.08)	

Ranges are posterior predictive probability intervals.

a Based on the number of samples analyzed: non-pome/non-thin, vehicle *n* = 41, house *n* = 31; non-pome/thin, vehicle *n* = 11, house *n* = 9; pome/non-thin, vehicle *n* = 25, house *n* = 23; pome/thin, vehicle *n* = 105, house *n* = 86.

b Based on the total number of samples: non-pome/non-thin, *n* = 47; non-pome/thin, *n* = 12; pome/non-thin, *n* = 29; pome/thin, *n* = 121; plus one sample with missing fruit classification.
